# Regulatable assembly of synthetic microtubule architectures using engineered microtubule-associated protein-IDR condensates

**DOI:** 10.1016/j.jbc.2024.107544

**Published:** 2024-07-09

**Authors:** Chih-Chia Chang, Scott M. Coyle

**Affiliations:** 1 Biophysics Graduate Program, University of Wisconsin-Madison, Madison, Wisconsin, USA; 2 Department of Biochemistry, University of Wisconsin-Madison, Madison, Wisconsin, USA

**Keywords:** cell circuits, cytoskeleton, microtubules, synthetic biology, condensates

## Abstract

Microtubule filaments are assembled into higher-order structures using microtubule-associated proteins. However, synthetic MAPs that direct the formation of new structures are challenging to design, as nanoscale biochemical activities must be organized across micron length-scales. Here, we develop modular MAP-IDR condensates (synMAPs) that enable inducible assembly of higher-order microtubule structures for synthetic exploration *in vitro* and in mammalian cells. synMAPs harness a small microtubule-binding domain from oligodendrocytes (TPPP) whose activity we show can be rewired by interaction with unrelated condensate-forming IDR sequences. This combination is sufficient to allow synMAPs to self-organize multivalent structures that bind and bridge microtubules into higher-order architectures. By regulating the connection between the microtubule-binding domain and condensate-forming components of a synMAP, the formation of these structures can be triggered by small molecules or cell-signaling inputs. We systematically test a panel of synMAP circuit designs to define how the assembly of these synthetic microtubule structures can be controlled at the nanoscale (*via* microtubule-binding affinity) and microscale (*via* condensate formation). synMAPs thus provide a modular starting point for the design of higher-order microtubule systems and an experimental testbed for exploring condensate-directed mechanisms of higher-order microtubule assembly from the bottom-up.

Microtubules (MTs) are one of the major filament systems of the eukaryotic cytoskeleton, extending throughout the cell to build up internal structure, position organelles at specific locations, and facilitate vesicle transport. Individual MTs are further organized into higher-order structures and machines critical for cellular processes, such as the mitotic spindle, ciliary axonemes, and diverse protozoan cytoskeletons (1, 2, 3, 4). The assembly and function of these structures depends on the action of MT-associated proteins (MAPs), which bind MTs and work across cellular length scales to direct the organization of a specific target structure (5, 6).

The diversity of MT-based structures and functions seen in nature suggests that synthetic MAPs could be engineered to assemble new MT structures with novel functions inside cells, analogous to how synthetic signaling proteins can rewire cellular information processing (7, 8, 9, 10). Such synthetic tools and strategies would not only be useful for cell-engineering applications but could also provide an experimental testbed exploring the general biochemical and biophysical mechanisms sufficient for directing the assembly of MT-based architectures from the bottom-up. However, because MTs are the largest filaments of the eukaryotic cytoskeleton, with a diameter of 25 nm and more than 1600 tubulin subunits per micron (11, 12, 13), it is not obvious how to best engineer synthetic MAP proteins such that their nanoscale activities can be effectively integrated across the microscale within cells.

Protein condensation mediated by phase separation has emerged as a mechanism by which nanoscale components can be organized and condensed into micron-scale droplets to drive biochemical interactions and chemistry in cells (14, 15, 16, 17). Although these droplets bear little resemblance to the ordered structures of the cytoskeleton, recent studies suggest roles for condensates in amplifying the activity of MAPs associated with MT bundling, branching, and tip growth such as tau, TPX2, NuMA, and +TIPs (18, 19, 20, 21, 22, 23). These observations are intriguing, as the high concentrations and reduced-order of condensates could provide the multimerization, valency, and flexibility needed to bridge length-scales (18, 19, 20, 21, 22, 23). However, these specific MAPs are not ideal candidates for synthetic manipulation and bottom-up explorations into sufficiency, as they are large multidomain proteins that work in multi-component networks to regulate structures essential for cell viability such as the mitotic spindle (24, 25, 26) (Fig. 1*A*).

**Figure 1:**
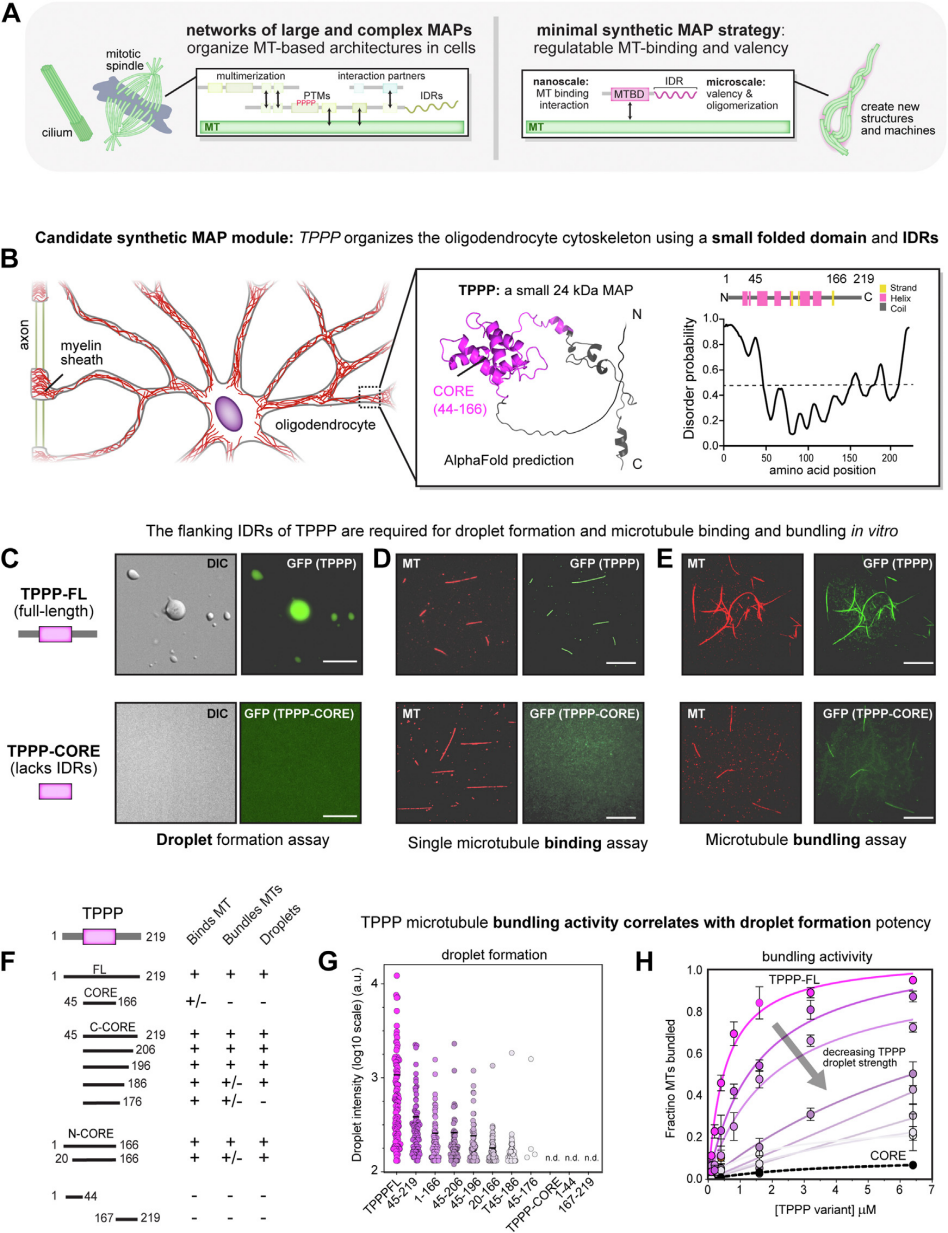
**Identification of TPPP as a condensate-regulatable microtubule bundling module for building synthetic microtubule assemblies.***A*, overview for designing synthetic microtubule-associated proteins (MAPs) to build new microtubule architectures. (*left*) Native MAPs, such as spindle assembly factors (*e.g.*, NuMA and TPX2), have multidomain architectures with many activities that provide valency, multimerization, and complex dynamic regulation; (*right*) a minimal design for a regulatable synMAP protein based on a single microtubule-binding domain to facilitate microtubule attachment and a condensate-forming sequence (IDR) to drive valency and multimerization. *B*, identification of TPPP as a candidate module for synthetic engineering using the strategy in (*A*). TPPP is a 24 kDa protein that promotes microtubule nucleation, bundling, and stabilization in oligodendrocytes. A 3D visualization of the AlphaFold structure prediction for TPPP is shown, with the folded CORE domain in *pink* and unstructured regions in *gray*. The corresponding secondary structure prediction of TPPP protein domains is indicated, with α-helical and β-strands structures are shown as *pink* and *yellow* blocks, along with the PrDOS-predicted intrinsic disorder of TPPP. Higher scores indicate a greater degree of disorder. *C*, epifluorescence images of TPPP droplet formation. TPPP, but not the isolated CORE domain, phase-separate into drops in the presence of 12% of dextran crowding agent. DIC (*left*) and *green* fluorescence (*right*) images of GFP-TPPP FL and GFP-TPPP-CORE, both at the concentration of 20 μM with 20 mM Hepes, 50 mM NaCl, 3 mM DTT, and 12% dextran. Scale bars represent 10 μm. *D*, TIRF images of single-microtubule binding for TPPP and isolated CORE domain. GMPCPP-stabilized microtubules (Alexa-594 and biotin-labeled), immobilized on a glass surface, were incubated with GFP-TPPP FL and GFP-TPPP-CORE, respectively, both at the concentration of 600 nM. Scale bars represent 10 μm. *E*, TIRF microscopy images of microtubule bundling for TPPP and isolated CORE domain. GFP-TPPP FL and GFP-TPPP-CORE (*green*) were mixed with *in vitro* polymerized Alexa-594 (*red*), biotin-labeled, and GMPCPP-stabilized microtubules for 5 min, then captured microtubule bundles by NeutrAvidin-coated biotin coverslips, both at the concentration of 6.4 μM. Scale bars represent 10 μm. *F*, behavior of different TPPP truncations and fragments with respect to MT-binding, bundling, and droplet formation activities. Symbols denote experiments performed qualitatively. *G*, quantification of GFP-TPPP variants from (*F*) with respect to droplet fluorescence signal intensity. The mean was determined from data pooled from five representative images. GFP-TPPP variants droplet number in total five images, respectively; TPPP FL, n = 107; TPPP -Ccore(45–219), n = 92; TPPP-Ncore(1–166), n = 73; TPPP(45–206), n = 53; TPPP(45–196), n = 60; TPPP(20–166), n = 89; TPPP(45–186), n = 90; TPPP(45–176), n = 4. TPPP-CORE(45–166), TPPP N-term, and TPPP C-term showed n.d., not detectable droplets. All GFP-TPPP variants were in the concentration of 20 μM with 20 mM Hepes, 50 mM NaCl, 3 mM DTT, and 12% dextran. N here refers to droplet number. *H*, MT bundling activity for the variants in (*F*) was quantified as an EC50 from full titration curves measuring the fraction of microtubules in bundles as a function of variant concentration. TPPP FL, EC50 = 0.54 μM; TPPP-Ccore, EC50 = 1.58 μM; TPPP-Ncore, EC50 = 2.06 μM; TPPP(45–206), EC50 = 10.58 μM; TPPP(45–196), TPPP(45–186), TPPP(20–166), and TPPP-CORE curves and EC50 constants were determined by fitting to a hyperbola. Mean (points) and SEM (error bars) of three replicate bundling experiments for each TPPP concentration is shown.

We sought a nonessential condensate-regulatable MAP with a simplified domain architecture that could provide a starting point for assembling and exploring synthetic MT architectures from the bottom-up. To this end, we focused on tubulin polymerization-promoting protein (TPPP). TPPP is a small 24 kDa MAP found in oligodendrocytes that contains only a single folded “CORE” domain (27, 28) (Fig. 1*B*). Despite its small size, TPPP is proposed to nucleate and stabilize MTs at discrete structures called Golgi outposts to promote elongation, branching, and polarization of myelin sheaths in oligodendrocytes (29). Pathologically, TPPP aggregates are associated with Parkinson’s disease and multiple system atrophy, and reversing TPPP aggregation has been proposed as a therapeutic strategy (30, 31, 32). Critically, the structured domain of TPPP is flanked by low-complexity intrinsically disordered sequences (IDRs), suggesting a possible role for protein condensation in its activity.

Here, we take advantage of TPPP’s minimal architecture and desirable features to develop synMAPs, engineered proteins that provide a regulatable platform for the assembly and synthetic exploration of higher-order MT structures *in vitro* and in living cells (Fig. 1*A*). Our designs are based on our discovery that TPPP’s native MT binding and bundling activity correlate with its intrinsic ability to form protein condensates. Both *in vitro* and in mammalian cells, we show that MT bundling activity can be synthetically rewired by fusing TPPP fragments to orthogonal condensate-forming IDR sequences completely unrelated to MT biology. This basic control logic can be exploited to engineer circuits that regulate the connection between TPPP and IDRs, allowing the assembly of large synthetic MT architectures to be triggered by small molecules and cell-signaling inputs. By systematically testing a panel of synMAP circuits that vary the strength of the MT-binding domain or the IDR, we experimentally define how synMAP-directed MT assembly can be biochemically tuned at both the nanoscale and microscale. synMAPs thus offer a compact modular starting point engineering synthetic MT architectures *in vitro* and inside living cells, as well as an experimental testbed for exploring the sufficiency of condensation mechanisms to facilitate higher-order MT assembly from the bottom-up.

## Results

### Identification of TPPP as a condensate-regulatable MT bundling module for building synthetic MT assemblies

TPPP is a small 24 kDa MAP found in oligodendrocytes that has been shown to bind and bundle MTs *in vitro*. Inspection of TPPP’s amino acid sequence and AlphaFold structure prediction revealed a folded ''CORE'' domain flanked by unstructured N- and C-terminal regions (IDRs) (Fig. 1*B*). As IDRs are common features of phase separating proteins, this suggested that TPPP might represent a compact condensate-regulatable starting point for engineering synthetic MAPs. To determine the relationship between TPPP’s IDRs and its biological activity, we applied three *in vitro* assays in parallel: 1) a droplet formation assay to determine whether a TPPP construct could phase separate in the presence of crowding reagents; 2) a TIRF-based assay for measuring binding to immobilized single MTs in the absence of crowders; and 3) a TIRF-based bundling assay that measures the fraction of MTs in bundles as a function of TPPP concentration to define an EC50 for quantitative comparison (detailed in Experimental procedures) (18, 24, 33).

We first expressed and purified GFP-tagged full-length TPPP and the isolated TPPP-CORE domain (aa.45–166), which completely lacks the unstructured N and C termini and tested each construct for droplet formation. We found that TPPP-FL phase-separated to form droplets under a wide range of different molecular crowding conditions (Fig. 1*C*). In fluorescence recovery after photobleaching (FRAP) experiments, TPPP-FL droplets showed clear fluorescent recovery over time, indicating dynamic exchange behavior (Fig. S1, *B* and *C* and Video S1). In contrast, no droplets were observed for TPPP-CORE under any conditions tested. Nearly identical results were observed using untagged constructs and DIC microscopy to visualize these droplets (Figs. 1*C* and S1*A*). TPPP can thus form protein droplets *in vitro* that depend on the presence of its flanking IDRs.

We then tested if TPPP droplet formation correlated with MT binding or bundling activity, measured in the absence of any molecular crowders. Droplet-competent TPPP-FL strongly decorated single-MTs immobilized on coverslips at concentrations as low as 600 nM, while the isolated CORE domain showed weak binding along MTs only at concentrations greater than 6 μM (Figs. 1*D* and S2). Similarly, while TPPP-FL showed strong MT bundling activity (EC_50_ = 0.54 μM), TPPP-CORE bundling was barely detectable even at concentrations greater than 6 μM (Fig. 1*E*). Thus, TPPP’s *in vitro* MT binding and bundling activity correlates with its ability to form droplets.

We next applied these assays to a series of TPPP truncations to quantitatively characterize the correlation between condensate formation and TPPP activity. Neither the N-terminal IDR (aa.1–44) nor the C-terminal IDR (aa.167–219) sequences were sufficient on their own to form droplets, bind, or bundle MTs (Figs. 1, *F* and *G* and S2*B*). However, when fused to the TPPP-CORE domain, both the N-terminal IDR (TPPP N-CORE) or the C-terminal IDR (TPPP C-CORE) could support droplet formation, MT binding, and MT bundling. Fine-scale truncations of the N-terminal IDR and C-terminal IDRs in this context showed that droplet size scaled with the length of the IDR extension (Fig. 1, *G* and *H*) and correlated with performance in our MT-bundling and single-MT binding assays (Figs. 1, *F*–*H* and S2*B*). Together, these data suggest that a TPPP construct’s bundling activity quantitatively correlates with its propensity (size and number) to form droplets.

### TPPP bundling activity can be rewired by fusion to orthogonal condensate-forming sequences to construct synMAP proteins

**Figure 2:**
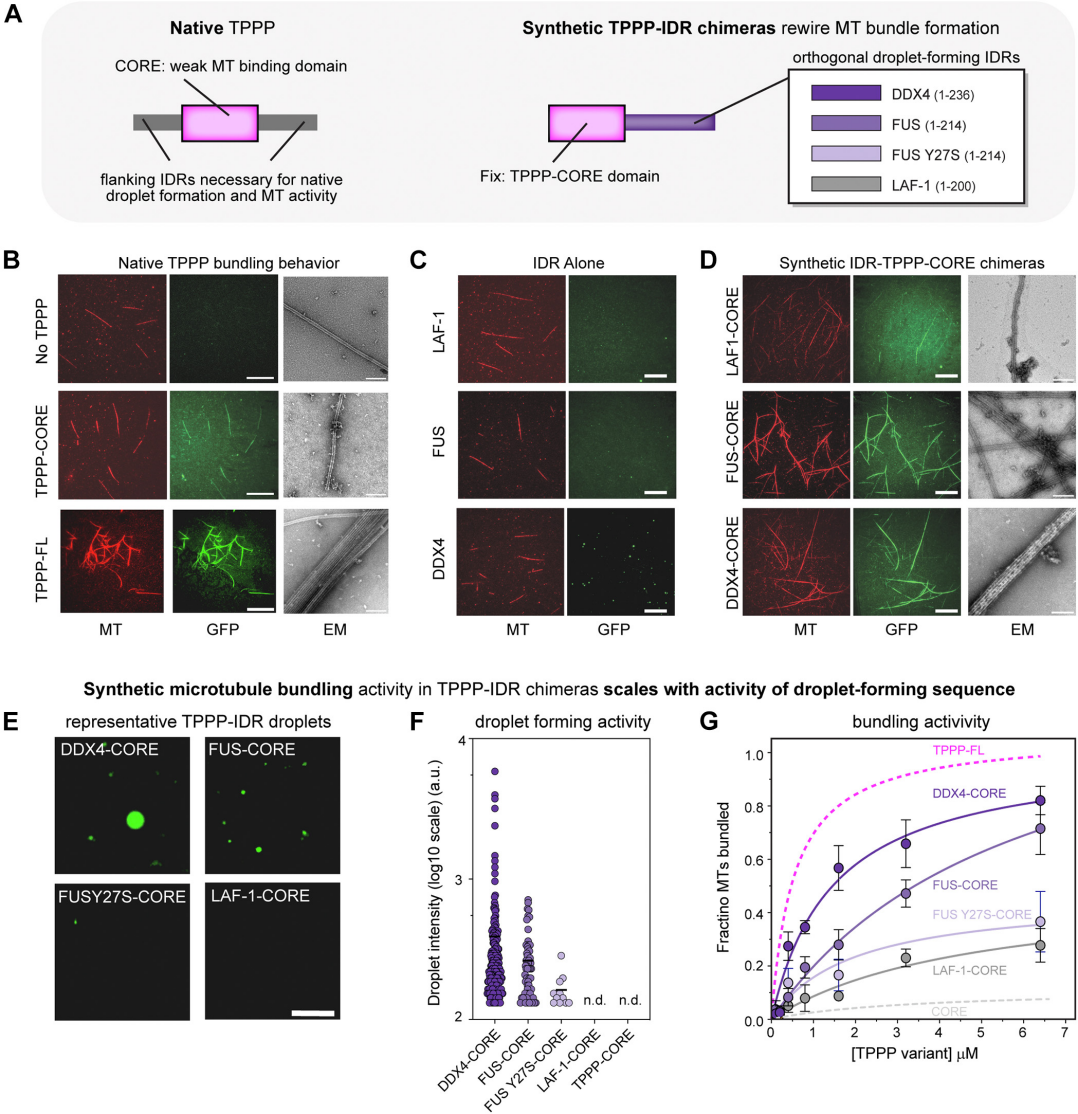
**TPPP bundling activity can be rewired by fusion to orthogonal condensate-forming sequences to construct synMAP proteins.***A*, strategy for rewiring TPPP activity using orthogonal IDR sequences to construct synMAPs. TPPP structured CORE domain (*left*) can act as a weak MT-binding domain but bundle formation requires the presence of one of the flanking intrinsically disordered regions (IDRs). Schematic of synMAP design (*right*): the IDRs of DDX4, FUS, FUSY27S (mutant), and LAF-1 were fused with TPPP-CORE domain as shown in table. These TPPP-IDR chimeras were tagged with GFP at their N terminus. *B*, TIRF and EM images showing the *in vitro* bundling behavior of native TPPP-FL and the isolated TPPP-CORE domain. Free microtubules are shown as a negative control. Scale bars represent 20 μm (TIRF images) and 200 nm (EM images). *C*, TIRF images showing that condensate-forming sequences (IDRs of DDX4, FUS, and LAF-1) do not interact with or bundle microtubules *in vitro*. Scale bars represent 20 μm. *D*, TIRF and EM images showing the *in vitro* bundling behavior of synMAP TPPP-IDR chimeras. Scale bars represent 20 μm (TIRF images) and 200 nm (EM images). *E*, representative fluorescent images of droplets formed by synMAP TPPP-IDR chimeras *in vitro*. Assays were performed at a protein concentration of 20 μM with 20 mM Hepes, 50 mM KCl, 3 mM DTT, and 12% dextran. Scale bars represent 10 μm. *F*, quantification of synMAP TPPP-IDR chimeras droplet fluorescence signal intensity. The mean was determined from data pooled from five different images. Droplet number was respectively: DDX4-CORE, n = 179; FUS-CORE, n = 57; FUSY27S-CORE, n = 11; LAF-1-CORE, n = 0; TPPP-CORE, n = 0. All synMAP TPPP-IDR chimeras were tested at the same concentration. N here refers to droplet number. *G*, MT bundling activity for the synMAP TPPP-IDR chimeras in (*F*) was quantified as an EC50 from full titration curves measuring the fraction of microtubules in bundles as a function of variant concentration. TPPP FL, EC50 = 0.54 μM, data was added as a point of comparison. DDX4-CORE, EC50 = 1.47 μM; FUS-CORE, EC50 = 5.92 μM; FUSY27S-CORE, and LAF-1-CORE curves and EC50 constants were determined by fitting to a hyperbola. Mean (points) and SEM (error bars) of three replicate bundling experiments for each TPPP concentration is shown.

Because the isolated TPPP-CORE domain has only weak MT-binding activity (Figs. 1*E* and S2*B*), we hypothesized that a function for condensates in this system could be to create the multimerization and high-valency needed to bridge multiple weak-binding events across multiple MTs into bundles. If true, it should be possible to engineer synthetic MAPs (synMAPs) that replace TPPP’s endogenous IDRs with alternative droplet-forming sequences completely unrelated to MT biology. To test, we designed and characterized synMAP TPPP variants which fused TPPP-CORE to well-characterized phase-separating IDRs: the IDRs of DDX4 (aa.1–236), FUS (aa.1–214) and LAF-1 RGG (aa.1–200) (34, 35, 36, 37, 38). These synMAP TPPP chimeras provide an orthogonal control knob for probing how arbitrary condensate formation can be harnessed to control and tune MT bundling activity (Fig. 2*A*).

On their own, the IDRs from DDX4, FUS, and LAF-1 were unable to bind or bundle MTs (Fig. 2*C*). However, fusion of these IDRs to the TPPP-CORE domain to generate synMAP proteins restored MT binding, bundling, and condensate formation activities (Figs. 2, *D* and *E* and S2, *C* and *D*). We quantified the droplet intensity distributions (Fig. 2*F*) and bundling efficiency (Fig. 2*G*) of these synMAPs and compared them to native TPPP-FL and truncations. Across synMAP IDR-CORE chimeras, bundling efficiency (EC50) scaled with droplet formation activity *in vitro* (DDX4>FUS>LAF). The strongest of these synMAPs, DDX4-CORE, had a MT-bundling activity (EC_50_ = 1.47 μM) within 3-fold of WT and outperformed many native bundling-competent TPPP truncations. In contrast, synMAPs based on LAF-1 and the mutant FUSY27S (39), which only weakly form droplets, showed weak MT-bundling activity (Fig. 2, *F* and *G*). Thus, synthetic modulation of TPPP condensate formation correlates with synMAP MT bundling activity analogous to its native IDRs.

While these assays show that synMAP TPPP-CORE-IDR fusions can stimulate MT bundling, they cannot resolve ultrastructural differences between the resulting bundled architectures. We thus examined the structure of native and synthetic TPPP MT bundles by negative stain electron microscopy. As has been reported previously, TPPP-FL bundles MTs into long arrays of tightly aligned MTs (26). In contrast, no bundling was observed for the condensate-deficient TPPP-CORE (Fig. 2*B*). synMAP IDR-CORE chimeras produced MT bundles in EM that agreed with their quantitative behavior in our *in vitro* bundling assays. The DDX4-CORE synMAP (EC_50_∼ 1.47 μM) produced bundles that closely resembled those seen for TPPP-FL, creating densely packed structures in which multiple MTs were aligned together (Fig. 2*D*). The weaker (EC_50_∼ 5.92 μM) FUS-CORE synMAP produced more disperse and less tightly-packed MT bundles (40) (Fig. 2*D*). No bundled structures were seen by EM for LAF-1-CORE synMAP, although some protein clusters were detected on the surfaces of single MTs (Fig. 2*D*).

Our results show that TPPP bundling activity can be synthetically controlled using alternative condensate-forming IDR sequences unrelated to MT biology *in vitro* to construct synMAP proteins. The correlation we observed between condensate formation and MT bundling across native and synthetic TPPP variants suggests that regulatable condensation could provide a mechanism for controlling synMAP MT bundling activity in cellular contexts.

### Synthetic clustering of TPPP triggers MT binding, but not bundling, in living cells

**Figure 3:**
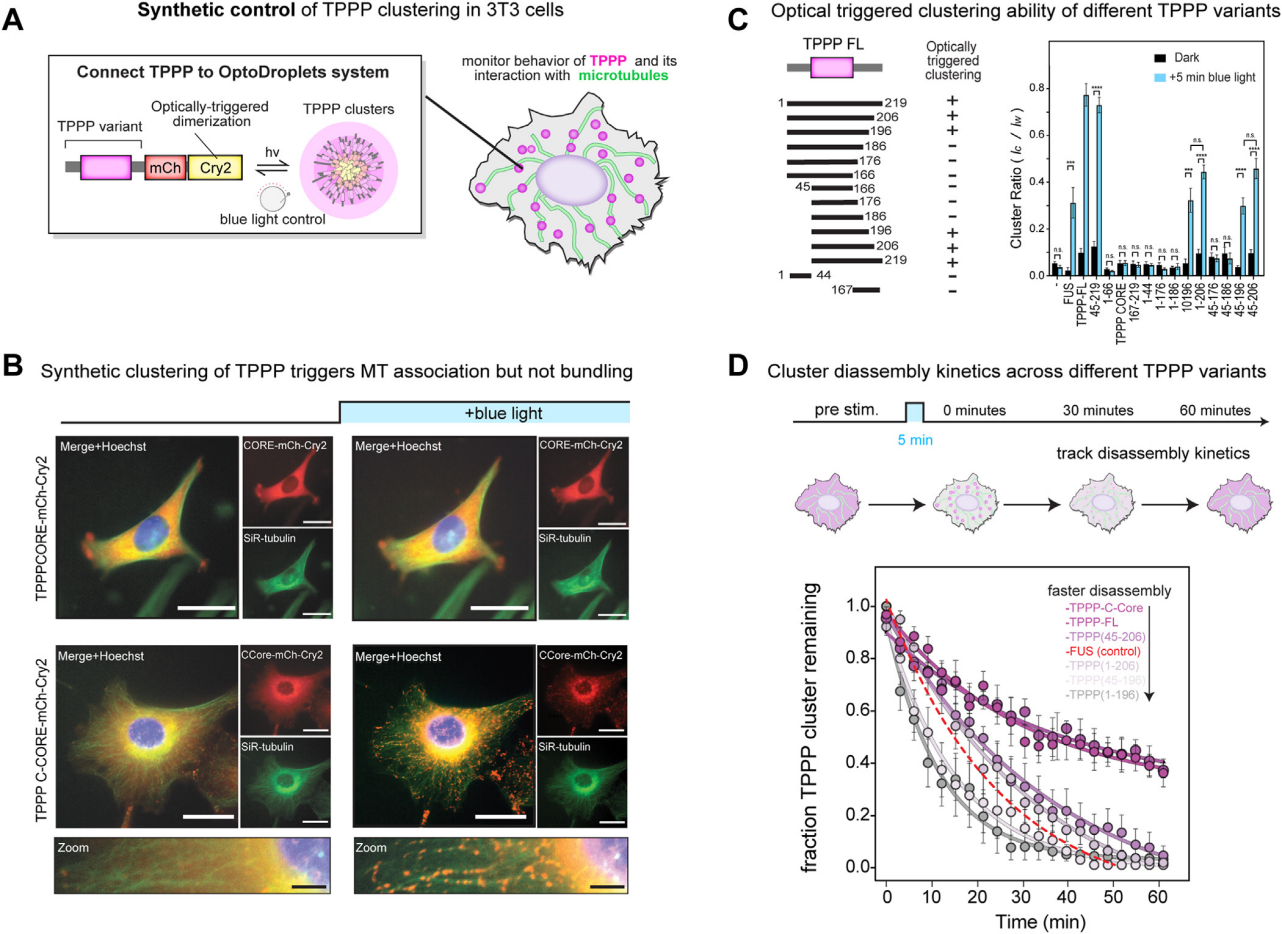
**Synthetic clustering of native TPPP fragments triggers microtubule binding, but not bundling, in cells.***A*, schematic diagram of constructs for blue-light triggered clustering of a TPPP fragment in cells by fusion to Cry2WT-mCh. *B*, representative images pre- and post- 5 min blue light activation for optical clustering of TPPP-CORE and the C-terminally IDR containing variant C-CORE in NIH3T3 cells (SiR-tubulin, *green*; Hoechst, *blue*). Scale bars represent 20 μm. For zoomed in insets of OptoTPPP C-CORE, scale bars represent 5 μm. *C*, schematic overview and quantification of light-induced cluster formation for different TPPP variants in cells. Clustering activity was scored by dividing cluster intensity (*I*_*C*_) by the total fluorescence (*I*_*W*_) of the whole cell. Values are expressed as means ± SEM. Cry2WT only, n = 12; FUS, n = 13; TPPP FL, n = 15; TPPPCcore(45–219), n = 15; TPPPNcore(1–166), n = 15; TPPP(1–206), n = 15; TPPP(45–206), n = 15; TPPP(1–196), n = 12; TPPP(45–196), n = 17; TPPP(1–186), n = 12; TPPP(45–186), n = 14; TPPP(1–176), n = 17; TPPP(45–176), n = 13; TPPPCORE(45–166), n = 12; TPPPN-term(1–44), n = 12; TPPPC-term(167–219), n = 12. Two-tailed Student *t* test; statistical differences: ∗∗∗∗, *p* < 0.0001; ∗∗∗ *p* < 0.001; n.s., not significant. N here refers to cell number. *D*, cluster disassembly kinetics in cells across different TPPP variants. Clusters were formed using a five-minute pulse of *blue* light. Following stimulation, the fraction of clusters remaining over time was tracked by normalization to this initial post-stimulation value. Values are expressed as means ± SEM. FUS, n = 6; TPPP FL, n = 6; TPPPCcore(45–219), n = 7; TPPP(1–206), n = 7; TPPP(45–206), n = 7; TPPP(1–196), n = 6; TPPP(45–196), n = 6. The curves were determined by fitting to a one-phase decay equation. N here refers to cell number.

Our *in vitro* results suggest that TPPP activity in living cells might be synthetically regulated by controlling when and where it is allowed to condense. To explore this systematically, we built a blue-light triggered TPPP clustering system in 3T3 cells to reconstitute TPPP activity from the bottom-up (Fig. 3*A*). Our approach is based on the established “OptoDroplets” system for optically triggering condensation of Cry2-IDR fusion proteins in living cells (41, 42). In this system, blue light triggers oligomerization of a Cry2-IDR fusion protein to increase the local concentration of its IDRs to a high enough level to drive droplet formation. We first validated this tool by expressing either mCh-Cry2WT or FUS-mCh-Cry2WT as a negative and positive control, respectively (Fig. S3*A*). As expected, mCh-Cry2WT showed no clusters upon light stimulation, while FUS-mCh-Cry2WT formed spherical drops distributed throughout the cell (39). Importantly, these controls showed no cross-reactivity of OptoDroplets with the MT cytoskeleton as visualized by the live-cell stain SiR-tubulin.

We then applied this tool to study the behavior of different TPPP fragments upon light-induced clustering. Prior to blue-light stimulation, droplet-competent TPPP-FL showed a diffuse distribution in the cell with only faint MT decoration. However, upon 5 min of blue-light stimulation, TPPP FL formed clusters that associated with and decorated the MTs lattice of the cell (Fig. S1, *B* and *C* and Video S2). In contrast, the condensate-deficient TPPPCORE-mCh-Cry2WT remained diffuse upon blue-light stimulation and no cluster formation was observed (Fig. 3*B* and Video S3). Large TPPP fragments can thus be synthetically clustered to trigger association with MTs, but this does not appear to give rise to extensive bundling in a cellular context (42).

Applying this opto-clustering method to the panel of TPPP fragments we tested *in vitro*, we found that C-terminal TPPP fragments showed robust cluster-induced MT association in living cells. In contrast, N-terminal TPPP fragments showed no light-dependent clustering onto MTs (Figs. 3*B* and S3*B*). Although these N-terminal fragments showed bundling and binding activity *in vitro*, their activity was weaker than C-terminal fragments. This suggests that the requirements for TPPP interaction with MTs in a living cell may be more stringent than in a minimal *in vitro* system. From this, we defined a minimal fragment (TPPP 45–196) that retains cluster-dependent MT association for synthetic applications (Fig. 3*C*).

This experimental setup also allowed us to investigate how the lifetime of light-induced TPPP clusters was affected by different TPPP fragments. We stimulated cells with blue light for 5 min to assemble TPPP clusters and then monitored cluster disassembly over time in in the dark (Figs. 3*D* and S4). Consistent with previous reports, optically induced FUS-mCherry-Cry2 control droplets completely disassembled within 30 min in the dark (41). In contrast, optically clustered TPPP-FL showed slow disassembly, with almost half of all TPPP clusters remaining after 1 h (Figs. 3*D* and S4). Applying this technique to our panel of TPPP variants, we found TPPP disassembly kinetics scaled inversely with *in vitro* droplet-formation activity. Collectively, these observations suggest that the condensate-forming activity of TPPP we observe *in vitro* can be harnessed to tune its cluster-induced association with MTs but that other mechanisms are required to reconstitute the formation of higher-order structures in living cells.

### synMAP TPPP-IDR condensates generate robust and biochemically tunable synthetic MT architectures in living cells

Our OptoDroplets experiments revealed that light-induced clustering of TPPP allows it to synthetically associate with MTs in cells. However, this approach did not produce the large-scale MT bundles observed *in vitro* and in the oligodendrocyte cytoskeleton. We noted that the number of TPPP clusters generated using the OptoDroplets system was high and potentially too finely dispersed to coalesce into higher-order architectures on the timescales accessible to light-stimulation. By comparison, the proposed mechanism for controlling TPPP activity in oligodendrocytes depends on its targeting to a recently discovered class of discrete and highly localized cell-type–specific structures called Golgi outposts (29, 43, 44, 45). These outposts are thought to act as hubs that concentrate TPPP with interaction partners, including the condensate-forming Golgi matrix protein GM130 (29, 46, 47).

**Figure 4:**
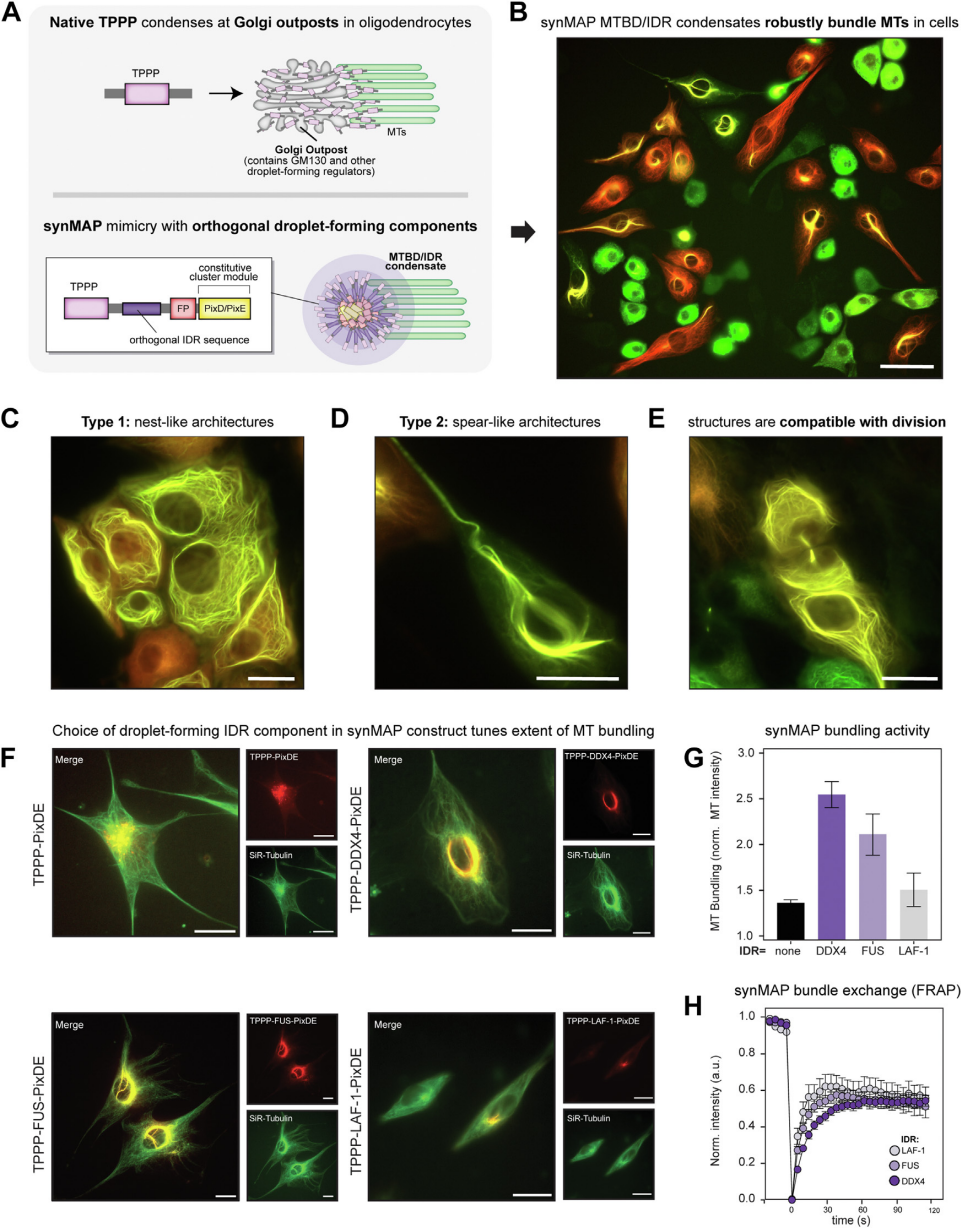
**synMAP TPPP-IDR condensates generate robust and tunable synthetic microtubule architectures in living cells.***A*, schematic diagram for the design of synMAP TPPP-IDR-FP-PixDE constructs. These are engineered to mimic the discrete and localized targeting of native TPPP to Golgi outposts in oligodendrocytes. *B*, representative image of a population of 3T3 cells expressing the TPPP-FUS-FusionRed-PixD and TPPP-FUS-Citrine-PixE synMAP design. SynMAPs condensates organize a diverse range of synthetic microtubule-bundled structures across various expression levels. Scale bar represents 60 μm. *C*, example image of a TPPP-FUS-PixDE synMAP driven nest-like MT architecture. Images are shown as a merge of the TPPP-FUS-FusionRed-PixD and TPPP-FUS-Citrine-PixE channels. Scale bars represent 20 μm. *D*, example image of a TPPP-FUS-PixDE synMAP-driven spear-like architecture. Images are shown as a merge of the TPPP-FUS-FusionRed-PixD and TPPP-FUS-Citrine-PixE channels. Scale bars represent 20 μm. *E*, example image showing a recently divided cell where a bundled MT-architecture has reformed at the site of separation, indicating synMAP structures are compatible with mitosis and cell division. Images are shown as a merge of the TPPP-FUS-FusionRed-PixD and TPPP-FUS-Citrine-PixE channels. Scale bars represent 20 μm. *F*, example epifluorescence images of microtubule architectures driven by synMAP TPPP-IDR condensates that differ in the identity of the condensate-forming IDR sequence. TPPP-PixDE (negative control), TPPP-DDX4-PixDE, TPPP-FUS-PixDE, and TPPP-LAF-1-PixDE images are shown as merged images of the synMAP (*red*) and SiR-tubulin (*green*) channels. Scale bars represent 20 μm. *G*, quantification of synMAP bundling activity generated by the constructs in (*F*), defined by the normalized intensity of SiR-tubulin signal near the cell center. Data for TPPP-PixDE (control) or different synMAP TPPP-IDR condensates are shown (mean ± SEM, TPPP-PixDE, n = 3; TPPP-DDX4-PixDE, n = 3; TPPP-FUS-PixD, n = 3; TPPP-LAF-1-PixDE, n = 3). N here refers to cell number. *H*, quantification of exchange dynamics within different synMAP-driven MT bundles, derived from FRAP recovery curves acquired by confocal microscopy. Points within the curve reflect the mean ± SEM: TPPP-DDX4-PixDE, n = 13; TPPP-FUS-PixDE, n = 14; TPPP-LAF-1-PixDE, n = 14. The dynamic recovery curves are derived from nonlinear curve fitting based on the one-phase association equation model. Related to (Fig. S5) N here refers to the number of cells on which the FRAP was performed.

Inspired by these models, we hypothesized that we could synthetically drive TPPP MT bundling in cells by directly connecting it to synthetic droplet-forming hubs. To develop these hubs, we took advantage of previous work showing that PixD/PixE proteins from *Synechocystis* can form persistent, micrometer-sized liquid-like droplets when fused with IDR sequences (48, 49, 50). We hypothesized that fusion of TPPP and IDR-PixD/E sequences would generate a synMAP-IDR composite of MT-binding domains (MTBDs) and condensate-forming sequences (IDRs) that would facilitate MT bundling (Fig. 4*A*).

To explore this possibility, we used lentiviral transduction to deliver different TPPP PixE/D synMAP designs into 3T3 cells, allowing us to sample the resulting behavior across a wide range of different expression levels. Expression of TPPP alone did not lead to appreciable MT bundling in 3T3 cells, while expression of IDR-PixD/E alone produced only constitutive droplets dispersed all throughout the cell with no apparent MT association. However, connecting these two activities together using TPPP-IDR-PixD/E synMAP fusion proteins led to robust and constitutive assembly of large synthetic MT architectures (Fig. 4*B*). For the TPPP-FUSN-PixE/D synMAP, these composite structures adopted several visually striking geometries within the cell, including “nest” like structures encircling the nucleus that spread to the cell cortex and “spear” like structures that ran from the center of the cell outwards, threading into extensions of the cell’s overall geometry (Fig. 4, *C* and *D*). When confined in these narrow spaces, these synthetic architectures would often buckle and twist to adopt helical shapes. Importantly, formation of these structures was only observed in cells expressing both the PixD and PixE synMAP components, and higher expression appeared to lead to more dramatic bundled structures. Surprisingly, these synthetic MT architectures were compatible with cell growth and division, as they disassembled at the onset of mitosis and rapidly reassembled post cell division (Fig. 4*E* and Video S5).

Our modular synMAP PixD/E design enabled us to test the ability of other condensate-forming IDR sequences to support synMAP bundle formation in cells. We found that an IDR sequence was absolutely required for bundle formation, as TPPP-PixD/E fusions did not generate bundles. In contrast, DDX4, FUSN, and LAF-1 all generated synMAP-directed MT bundles in cells, albeit to differing degrees (Fig. 4*F*). To facilitate direct comparison and quantification, we used cell-sorting to enrich PixD/E-positive cells with similar expression levels. By quantifying the magnitude of MT bundling across multiple expression-matched cells within these sorted populations (detailed in Experimental procedures), we found that IDRs with stronger droplet-forming activity (size and number) led to more potent synMAP bundling activity (Fig. 4*G*). These bundles showed different exchange dynamics, as judged by their FRAP recovery kinetics, with weaker IDR sequences showing faster exchange (Figs. 4*H* and S5). Taken together, our results indicate that engineered synMAPs that fuse TPPP to constitutive droplet-forming activity provide a simple and biochemically tunable means of generating artificial and biologically-compatible MT bundles in mammalian cells.

### Engineered circuits can trigger the formation of synthetic MT architectures by regulating the connection between synMAP’s TPPP and IDR components

**Figure 5:**
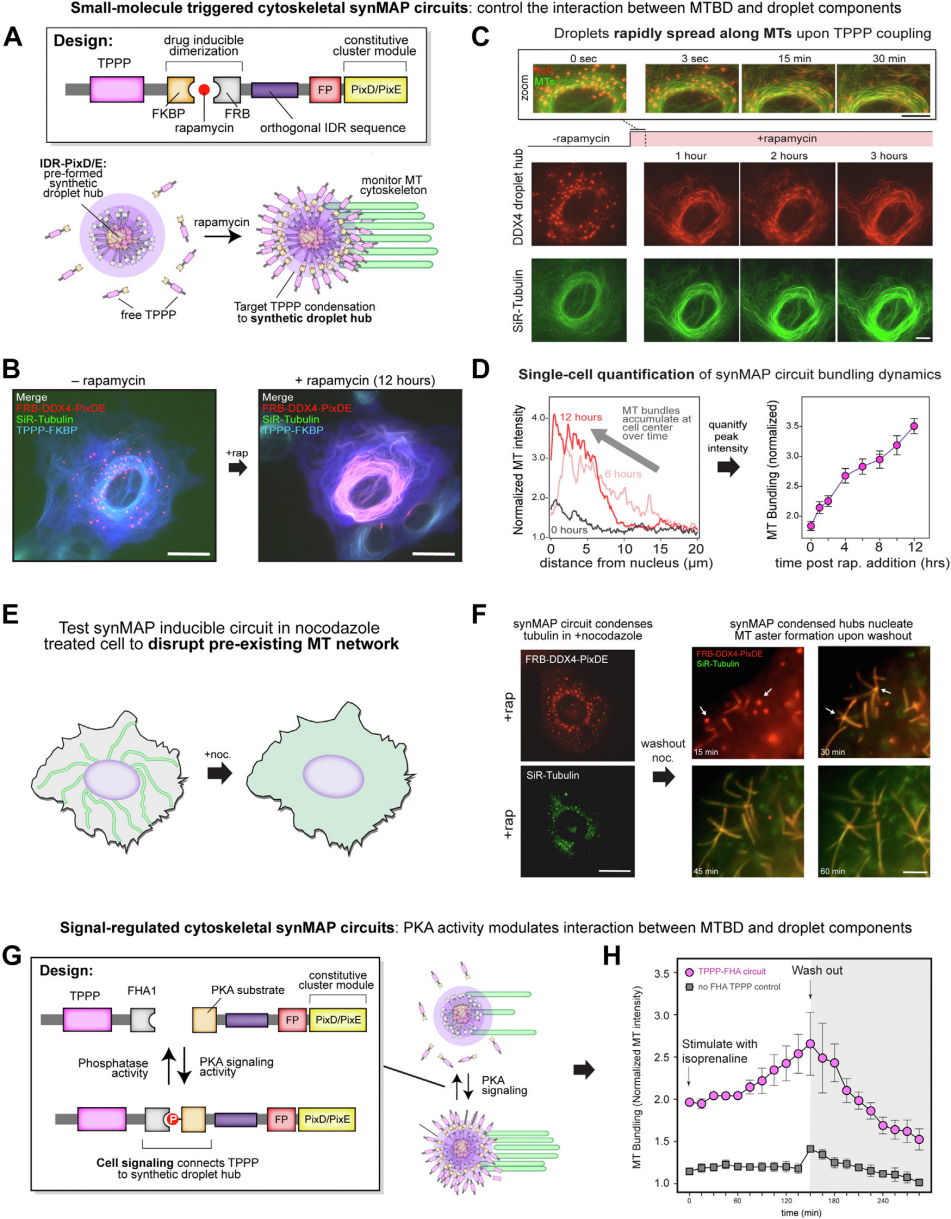
**Engineered circuits can trigger synthetic microtubule architectures by regulating the connection between synMAP’s TPPP and IDR components.***A*, schematic of cytoskeletal circuit designs that inducibly connect TPPP to IDR-droplet hubs, mimicking the discrete and localized targeting of native TPPP to Golgi outposts in oligodendrocytes. The system is based on the rapamycin-inducible dimerization pair FKBP/FRB. FRB-IDRs are fused to PixD/E-containing fluorescent proteins (FusionRed and Citrine) to produce constitutive droplets, and FKBP is fused to TPPP tagged with tagBFP. Upon rapamycin treatment, a synMAP’s MT binding and IDR components become connected to stimulate the assembly of higher-order MT structures. *B*, representative images of NIH3T3 cells cotransduced with an inducible synMAP circuit (FRB-DDX4-PixDE and TPPP-FKBP) before and after (12 h) rapamycin treatment. The addition of rapamycin (20 μM) rapidly translocated the TPPP component (tagBFP, *white* color in image) into DDX4 condensates (FusionRed, *red*) to initiate microtubule bundling (SiR-tubulin, *green*). Scale bar represents 20 μm. *C*, images from a time series of the rapamycin-induced synMAP circuit from (*B*). Microtubule lattices were directly visualized with SiR-tubulin (*green*). Zoom view (*upper*) shows the time series for early stages (0–30 min) post rapamycin induction, in which connection of TPPP fragments to condensates causes deformation and wetting of synMAPs onto microtubules. Over several hours, these structures coalesce into larger bundled architectures that resemble those formed by synMAPs that directly fuse the IDR and MT interacting components (*bottom*). Scale bar represents 10 μm. (see also Video S4). *D*, quantification of the rapamycin-inducible synMAP circuit in (*B*)-(*C*) over time. (*left*) Line profiles showing MT distribution (normalized SiR-tubulin fluorescence) as a function of the distance from the cell center reveal how microtubules are organized into bundled architectures near the cell-center over time. The line profile for each time point is used to generate a trajectory (*right*) showing the single-cell MT bundling dynamics (normalized intensity of SiR-tubulin signal at the cell center) generated by the synMAP circuit over time. Data are shown as (mean ± SEM, n = 24 cells). *E*, schematic for studying synMAP circuit dynamics following nocodazole-induced microtubule depletion. *F*, inducible synMAP circuits (TPPP-DDX4 design) can nucleate microtubule aster formation following nocodazole treatment as shown in (*E*). (*left*) Representative image showing SiR-tubulin colocalization with synMAP (FusionRed, *red*) droplets in cells treated with nocodazole (30 μM) for 6 h. No microtubules are present at this time. Scale bar represents 20 μm. (*right*) Live-cell images of synMAP circuit behavior following nocodazole washout. Images show microtubule aster formation (*arrows*) originating from synMAP TPPP-DDX4 condensates over time. Scale bar represents 5 μm. *G*, schematic for a synMAP circuit in which microtubule architecture is regulated by PKA signaling activity. A PKA substrate was fused to the condensate-forming DDX4-PixDE component and co-expressed with a TPPP-FHA1 (binds phosphorylated PKA substrate) or TPPP (negative control) component in NIH3T3 cells. When the PKA substrate is phosphorylated in response to cell-signaling inputs, TPPP will be connected to the IDR component to stimulate synMAP circuit activity. *H*, demonstration of the PKA-regulated synMAP circuit design from (*G*). Cells were stimulated with isoprenaline and imaged for 135 min. Isoprenaline was then washed out and the cells were further imaged for another 135 min. The circuit’s bundling dynamics were quantified as in (*D*). Data are shown as (mean ± SEM, PKAsub-DDX4-PixDE/TPPP-FHA1, n = 3; PKAsub-DDX4-PixDE/TPPP, n = 3). N here refers to cell number.

Our above results show that synMAPs that directly fuse TPPP to droplet-forming sequences drive constitutive assembly of higher-order MT architectures in cells. Thus, we reasoned that synMAPs could be used to build more complex cytoskeletal circuits by separating the TPPP and IDR components and placing their physical connection with one another under inducible control. To test this idea, we fused one half of the rapamycin-dependent dimerization system FRB to the IDR-PixDE component and fused the other half FKBP to the TPPP component (51, 52). This synMAP circuit design should enable TPPP to be targeted to pre-formed droplet hubs upon rapamycin addition and resultantly trigger formation and assembly of synthetic MT architectures (Fig. 5*A*).

We first examined the performance of a synMAP circuit targeting TPPP-FL-FKBP to FRB-DDX4-PixDE droplet hubs in 3T3 cells. Prior to the addition of rapamycin, TPPP FL-FKBP was distributed throughout the cytoplasm and partially decorated MTs at the nuclear periphery (53). Strikingly, rapamycin-induced targeting of TPPP-FL-FKBP to FRB-DDX4-PixDE droplet hubs triggered assembly and extensive MT bundling within the cell (Fig. 5*B*). Detailed inspection of this process revealed that droplet hubs were the initial sites of MT interaction (Fig. 5*C*). Droplets began to deform within seconds of TPPP coupling and completely spread across the MT surface within 15 min. Over 12 h, interactions between adjacent TPPP-coated MTs resulted in extensive redistribution of the cell’s MT network into nest-like bundles in which the SiR-tubulin signal increased >3 fold relative to the start of the experiment (Fig. 4, *C* and *D* and Video S4). Thus, inducible synMAP circuits targeting TPPP to a DDX4-droplet hub allowed temporal triggering of the formation of synthetic bundle architectures.

TPPP has also been proposed to play a role in the nucleation of MTs at Golgi outpost hubs in oligodendrocytes (29, 44, 54). However, in 3T3 cells, MT nucleation through the centriole dominates the cell’s interphase MT network. Thus, to test whether synMAP circuits that target TPPP to a droplet-hub could trigger aster formation, we first treated cells with nocodazole to depolymerize all MTs (Fig. 4*E*). We then targeted TPPP to PixDE-DDX4 droplets using rapamycin and performed a wash-out experiment (54). Prior to wash-out, TPPP and tubulin strongly colocalized with DDX4 droplet hubs, but no MTs were detected owing to the presence of nocodazole (Fig. 4, *E* and *F*). However, upon nocodazole wash-out, MT filaments rapidly emerged from many TPPP droplet-hubs in an aster-like structure (Fig. 4*F* and Video S6). Over time, multiple droplet-asters clustered and fused together to form MT bundles that coalesced into nest-like architectures as described earlier (Fig. S6 and Video S6). This result indicates that synMAP circuits can stimulate and stabilize the nucleation of new MTs when sufficiently isolated from other sources of nucleation and polymerization.

Next, we tested whether the same design principles governing the FKBP/FRB synMAP circuit could be used to trigger the formation of MT architectures in response to endogenous cell-signaling activity, using PKA signaling as a test case. To this end, we replaced the FKBP/FRB heterodimerization module with an FHA/PKAsub interaction pair, in which the FHA domain only binds to PKAsub when it is phosphorylated by PKA (55, 56). Expression of this TPPP-FHA1/PKAsub-DDX4-PixD/E synMAP circuit led to moderate levels of constitutive bundling in unstimulated cells, consistent with some basal interaction between the FHA1 and PKAsub components (57) (Fig. 5*G*). Stimulation of these cells with a saturating dose of the PKA agonist isoprenaline led to a quantitative increase MT bundling over 2 h and decreased back to baseline levels within 1 h following wash out (Fig. 5*G* and Video S7). In contrast, a TPPP/PKAsub-DDX4-PixD/E negative control circuit that lacks the FHA1 interaction module showed neither baseline nor inducible MT bundling (Figs. 5*G*, S7).

Together, these synMAP circuits demonstrate that the formation of a synMAP MT architecture can be dynamically turned on or off by regulating the interaction between its MT binding component (TPPP) and its droplet-forming component (IDR-PixD/E). These results suggest synMAPs could be used as modules for even more complex or elaborate circuit designs in mammalian cells going forward.

### MT interaction strength and IDR potency provide two levels of control for inducible synMAP bundling circuits in cells

**Figure 6:**
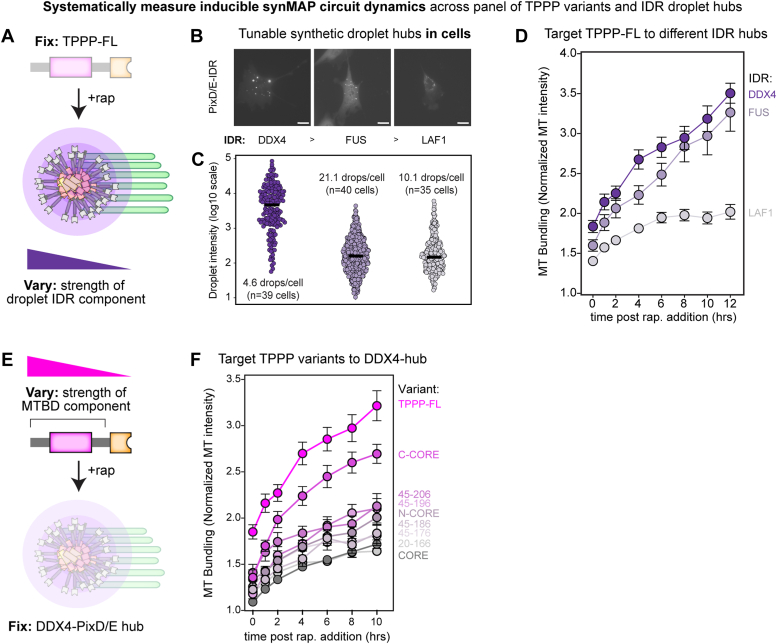
**Microtubule interaction strength and IDR potency provide a two-level control scheme for tuning inducible synMAP bundling circuits in cells.***A*, schematic depicting strategy for systematically varying the condensate-forming component (IDR sequence) of an inducible synMAP TPPP-IDR circuit. *B*, representative images from NIH3T3 cells (FusionRed, *white* color in images) showing the size and distribution of the IDR-droplet component of the different synMAP circuits. Scale bar represents 20 μm. *C*, quantification of the IDR-droplet components from (*B*), based on FusionRed fluorescence signal intensity in NIH3T3 cells. Median intensity is indicated from measurements from 39, 40, and 35 representative cells, with total droplet numbers respectively: FRB-DDX4-PixDE, n = 182; FRB-FUS-PixDE, n = 843; FRB-LAF-1-PixDE, n = 354. From these data, an average number of droplets per cell was also determined as follows: FRB-DDX4-PixD = 4.1 drops/cell, FRB-FUS-PixDE = 21.1 drops/cell, and FRB-LAF-1-PixDE = 10.1 drops/cell. N here refers to droplet number. *D*, quantification of synMAP circuit induction for the panel of IDR-varying designs from (*A*), using the method described in Fig. 5*D*. Data are shown as (mean ± SEM, FRB-DDX4-PixDE/TPPP-FL-FKBP, n = 24; FRB-FUS-PixDE/TPPP-FL-FKBP, n = 27; FRB-LAF-1-PixDE/TPPP-FL-FKBP, n = 31). Related to (Fig. S8). N here refers to cell number. *E*, schematic depicting strategy for systematically varying the microtubule-interaction component (TPPP variant) of an inducible synMAP TPPP-IDR circuit. *F*, quantification of synMAP circuit induction for the panel of TPPP-varying designs from (*E*), using the method described in Figure 5*D*. Data are shown as (mean ± SEM, TPPP-FL-FKBP/FRB-DDX4-PixDE, n = 24; TPPPCcore-FKBP/FRB-DDX4-PixDE, n = 16; TPPPNcore-FKBP/FRB-DDX4-PixDE, n = 15; TPPP(45–206)-FKBP/FRB-DDX4-PixDE, n = 12; TPPP(45–196)-FKBP/FRB-DDX4-PixDE, n = 12; TPPP(45–186)-FKBP/FRB-DDX4-PixDE, n = 18; TPPP(45–176)-FKBP/FRB-DDX4-PixDE, n = 26; TPPP(20–166)-FKBP/FRB-DDX4-PixDE, n = 15; TPPP-CORE-FKBP/FRB-DDX4-PixDE, n = 36). Related to (Fig. S9). N here refers to cell number.

In the above synMAP circuits, we found that inducibly targeting TPPP to a DDX4 droplet hub triggered MT bundling activity, which could be quantified by tracking the accumulation of MTs towards the perinuclear region of the cell over time. These metrics allow us to take a systematic approach to experimentally define how bundling dynamics of a synMAP circuit are quantitatively affected by either 1) the strength of the TPPP MT binding component or 2) the strength of the droplet-forming IDR component (Fig. 6, *A* and *E*).

To compare the effect of different IDR droplet-hubs, we first expressed different FRB-IDR-PixDE constructs in 3T3 cells and characterized the size, number, and exchange dynamics of the resulting hubs. We found that FRB-DDX4-PixDE created the greatest number of large droplets (>1000 a.u.) in cells, followed by FRB-FUS-PixDE (large number of small droplets) and FRB-LAF-1-PixDE (small number of small droplets) (Fig. 6, *B* and *C*). We then built synMAP trigger circuits targeting TPPP-FL to each class of IDR droplet-hub and quantified bundle formation at the single-cell level across multiple replicate sorted and expression-matched cells (Fig. 6, *A* and *D*). When TPPP-FL was targeted to DDX4 droplet-hubs, the MT-bundling triggered was strong, producing the highest density of MTs in bundles over a 10-h period. FUS droplet-hubs also produced substantial MT bundling, showing a similar rate of bundling as DDX4 but with slightly reduced basal activity and a lower end-point. In contrast, the weaker droplet-forming LAF-1 hubs showed significantly lower bundling rates and lower end-point bundling (Figs. 6*D* and S8). These synMAP circuit dynamics correlate with the droplet-formation strength (size and number) of the IDR-PixD/E module (DDX4>FUS>LAF1) (Fig. 2), indicating that synMAP circuit behavior in cells can be tuned by manipulating the microscale properties of the condensate component.

We next tested the effects of manipulating the strength of the TPPP MT binding component on synMAP trigger circuit behavior (Fig. 6*E*). Having seen that DDX4 droplet-hubs had the most striking bundling phenotypes, we locked in this component of the circuit and targeted TPPP fragments with different interaction strength (as defined by our *in vitro* and optoDroplets analyses) to these hubs. We found that TPPP variants with strong MT-association triggered the strongest MT bundling activity when targeted to DDX4-droplet hubs (*e.g.* C-CORE) (Figs. 3*C*, 6*F*, S2*B*, and S9, *A*–*C*). Variants that could bundle MTs *in vitro* but showed poor activity in the OptoDroplets assay triggered limited bundling activity (*e.g.* N-CORE) (Figs. 3*C*, 6*F* and S9, *D*–*F*). Targeting TPPP-CORE to DDX4 droplets triggered weak, but detectable, MT bundling in this assay (Fig. S9, *G*–*I* and Video S8). However, the degree of bundling through TPPP-CORE is dramatically lower than what we observed *in vitro*. Indeed, the majority of TPPP-CORE remained in droplets when targeted to DDX4 hubs, in contrast to other TPPP variants that induced droplet-spreading along the MT surface. This suggests that although a droplet hub can enhance a weak MT-binding activity, if droplet formation is too strong relative to MT-binding, it may outcompete the target MT interaction in cellular contexts.

Taken together, these data clarify how two key biochemical parameters of a synMAP design control the resulting triggering circuit’s ability to induce MT bundling. Stronger MT interaction strength allowed for some synMAP-driven bundling even in the absence of direct targeting to a droplet hub. Bundling activity dramatically increased across all variants when targeted to a strong droplet hub (DDX4), but TPPP fragments with reduced MT interaction strength were less effective. Together, this implies two natural routes for achieving spatiotemporal control of synMAP circuit activity in cells going forward: modulation at the nanoscale, targeting how strongly TPPP can engage MTs; or modulation at the microscale, targeting the condensate-formation activity of the IDR component.

## Discussion

We have identified and exploited a condensate-regulatable MT-binding activity from the oligodendrocyte protein TPPP to develop synMAPs: engineered proteins that provide inducible control over higher-order MT structure formation *in vitro* and in mammalian cells. The central design feature of synMAPs is the pairing of TPPP’s native MT-binding activity with other orthogonal, condensate-forming IDR sequences that are unrelated to MT biology. *In vitro*, direct fusion of IDRs to TPPP’s minimal structured domain stimulates potent MT bundling that quantitatively correlates with the droplet-forming activity of the IDR. In living cells, synthetic MT bundling requires the connection of larger TPPP fragments to constitutive droplet-forming sequences. We capitalized on this stringency to engineer cytoskeletal circuits in cells in which synMAP activity could be induced by regulating the interaction between the MTBD and droplet-forming components. This allowed the formation and dynamics of MT architectures to be triggered by small molecules and endogenous cell-signaling activities. By systematically exploring and analyzing synMAP circuit behavior using different MTBDs and droplet-forming sequences, we identify biochemical strategies for controlling synMAP-triggered assembly of MT architectures at both the nanoscale and microscale.

A surprising result that emerged from our synthetic approach is that many critical cytoskeletal activities—MT binding, bundling, and aster formation—could all be driven and regulated through the action of arbitrary droplet-forming sequences which have not specifically co-evolved with MTs to perform these functions. Indeed, the ability of TPPP’s MT-binding domain to cooperate so effectively with the IDRs of DDX4—an RNA helicase (58, 59)—indicates considerable plasticity in the mechanisms by which condensate-formation can synergize with MT organization. We suspect this flexibility may explain the multitude of MT structures of other TPPP isoforms are associated with outside of oligodendrocytes, including the sperm flagellum (TPPP2) (27, 60) and protozoan ciliary lattices (61, 62). More generally, our results provide bottom-up support for previously suggested hypotheses (18, 19, 22, 23) about roles for droplet-forming sequences in native MAPs: that droplet formation may provide a versatile *biophysical* strategy for coordinating the nanoscale interaction between MAPs and MTs across cellular length scales (Fig. 6*G*). By approaching this hypothesis from a synthetic biology perspective, our results circumvent the difficulties associated with disentangling condensation from other activities in highly evolved native proteins and provide evidence supporting the sufficiency of condensation as a general biophysical strategy for organizing MTs.

At the same time, it is critical to recognize that these general-purpose biophysical mechanisms must be paired with more sophisticated spatiotemporal regulatory mechanisms to reliably control the assembly of specific architectures and cellular functions. For example, while TPPP was able to potently bundle MTs within the cell, the interphase MT network derived from centriolar MTOCs dominated the overall structure and spatial distribution of the emergent structure towards the cell center (63, 64, 65). This limitation motivated our embedding of synMAPs in more complex cytoskeletal circuit designs, in which we introduced engineered switches mediated by small molecules or cell-signaling inputs to provide *temporal* control over when bundling would be triggered.

Extending these approaches to allow more precise, titratable regulation over synMAP activity in the future will be needed to generate and control more complex MT structures. For example, dose-dependent titratable regulation of synMAP activity would allow MT assemblies to dynamically adapt and respond to different levels of stimulus. Similarly, spatially localized synMAP activity or activity gradients might allow more precise structural organization and positioning of synthetic MT architectures within the cell. Although we do not achieve such regulation at present, the modular design of synMAPs allows their activity to be triggered by reassembling the MT binding and droplet-forming components, suggesting more complex modes of regulation could likely be achieved through connection to other synthetic biology or chemical biology tools. Nevertheless, our results show that droplet-based biophysical mechanisms provide a tractable starting point for the design of higher-order MT-based cellular structures, providing a much-needed tool for synthetic biology to begin tackling the challenge of engineering the MT cytoskeleton of living cells in earnest.

The synMAP circuits we present generate new cytoskeletal structures inside cells, but these structures do not yet encode new biological functions. However, our droplet-based design provides straightforward paths going forward to further equip these structures with additional activities based on co-condensation of IDR-matched client proteins into the structure (14, 66). Such strategies could be used to selectively recruit specific motor proteins to generate bending forces and motion or to localize and position biochemical cargos or protein payloads and activities within the cell. By synthetically building these structures and systems from the ground up, synMAPs have the potential to reveal general principles underlying the diversity of complex MT architectures seen in nature and a path forward to engineering new MT structures or machines of our own design.

This manuscript includes a Supplemental Information file.

## Experimental procedures

### Plasmid construction

*Homo sapiens* TPPP WT gene was synthesized as gBlocks (Integrated DNA Technologies). TPPP truncation mutants were produced using a standard PCR. All TPPP variants were then inserted into a pBH4 vector which contains GFP sequence and His-tag at N-terminal region. *H. sapiens* DDX4 (residues 1–236), LAF-1 RGG (residues 1–200), and FUS Y27S mutant (residues 1–214) fragments were obtained as gBlocks (Integrated DNA Technologies). Human FUS (residues 1–214) was amplified by PCR using pHR-FUSN-mCh-Cry2WT (Addgene #101223). The IDR fragments were fused with TPPPCORE domain(aa.45–166) into the same pBh4-His-GFP plasmid. For making the OptoTPPP, different TPPP fragment were produced by PCR then cloned into the pHR-mCh-Cry2WT plasmid.

The DNA fragments of FRB, IDRs, FusionRed-PixD, and Citrine-PixE were produced by PCR using existing templates (Addgene #31181), (Addgene #111503) and (Addgene #111505) and subcloned into an SFFV vector to generate FRB-IDR-FusionRed-PixD and FRB-IDR-Citrine-PixE. The DNA fragments of TPPP FL and FKBP were produced by PCR using existing templates and (Addgene #31184). The tagBFP gene was synthesized as gBlocks (Integrated DNA Technologies). These fragments were subcloned into an SFFV vector to generate TPPP-tagBFP-FKBP. For making different TPPP variants-tagBFP-FKBP constructs, the DNA fragments were generated by PCR and then fused into the SFFV promoter vector.

To create the TPPP-tagBFP-FHA1 and PkAsub-DDX4-FusionRed-PixD/Citrine-PixE constructs, we first fused the PKA substrate sequence "LRRATLVD" to the N-terminus of DDX4-FusionRed-PixD and DDX4-Citrine-PixE, resulting in the PKAsub-DDX4-FusionedRed-PixD and PKAsub-DDX4-Citrine-PixE constructs. Next, we generated the FHA1 gene *via* PCR using existing templates (addgene #138202). This FHA1 gene was then introduced to replace the FKBP sequence in the TPPP-tagBFP-FKBP construct, resulting in the creation of the TPPP-tagBFP-FHA1 construct. All fragments were subcloned to the SFFV promoter vector.

### Bacterial protein expression and purification

All TPPP variant and TPPP-chimeras constructs were expressed in BL21(DE3). *Escherichia coli* overnight at 16 °C after induction with 0.5 mM IPTG. The culture was harvested by centrifugation and lysed according to the following procedure. The cell pellets were resuspended in lysis buffer (50 mM KH2PO4, 50 mM Na2HPO4, 150 mM NaCl, and 2 mM β-mercaptoethanol), disrupted by French press, and centrifuged at 15,000*g* at 4 C for 20 min. The supernatant was incubated with Ni resin (Sigma) for 30 min, followed by a prewash with 25 mM imidazole. Proteins were eluted with the buffer containing 250 mM imidazole. Then the samples were further purified by SEC (Superdex 200 16/60) and analyzed by SDS-PAGE. The peak protein fraction was collected, concentrated, and stored in a buffer consisting of 20 mM Hepes, pH 7.4, 50 mM NaCl, and 3 mM DTT. All proteins were flash frozen and stored at −80 °C. Before use, all proteins were pre-cleared of aggregates *via* centrifugation at 4 °C.

### Single-MT binding and MT-bundling assays

MTs were polymerized from a solution that contained unlabeled, Alexa Fluor 594-labeled and biotin-labeled tubulin in a ratio of 25:1:1 at 37 °C in the presence of GMPCPP. Flow chambers were assembled with biotin-PEG–coated coverslips. The chamber was sequentially filled with 0.5 mg/ml α-casein, 0.2 mg/ml NeutrAvidin, and biotinylated MTs in 1xBRB80 and 20 μM Taxol. Next, GFP-labeled TPPP variants or TPPP-Chimeras were added to the chamber in 1xBRB80 supplemented with 20 μM Taxol, 0.5 mg/ml α-casein, 10% sucrose, 2 mM DTT, 200 μg/ml glucose oxidase, 35 μg/ml catalase, and 4.5 μg/ml glucose. The chamber was then filled with the solution and sealed, then imaged by using TIRF microscopy. The GFP and Fluor 594 intensities were determined using Fiji.

For a MT bundling assay, TPPP variants and TPPP chimeras were incubated with Taxol-stabilized, Alexa Fluor 594-labeled and biotin-labeled tubulin MTs for 5 min. The mixture of bundled and free MTs was captured onto a biotinylated coverslip for quantitative determination of the fraction of MTs bundled. MT bundles were visualized by a TIRF illumination system, and Fluor 594 intensities were determined using Fiji. Single MTs and bundled MTs were distinguished by the fluorescence intensity line profile along the structure, using the intensity distribution from an unbundled, single MT sample as a reference. A bundled structure was defined as any structure with intensity greater than two SDs above the mean-value for the single MT distribution. Using this metric, the bundle-fraction for a given condition was defined as the fraction of MT structures observed that were classified as bundles in the sample. This method was used to produce all saturation curves, in which a concentration series was performed for each TPPP variant or TPPP-Chimeras. EC50s were obtained by fitting these curves to a one-site–binding hyperbola (24, 33, 67).

### Droplet formation assay

GFP-TPPP variants and TPPP-chimeras with 20 μM in buffer (20 mM Hepes, 50 mM NaCl, 3 mM DTT) containing crowding reagents (12% dextran), respectively. Samples (2 μl) were spotted onto glass slides then visualized using Confocal fluoresce microscopy. Images were visualized GFP signal, then measured and quantified using Fiji.

### FRAP analysis

GFP-TPPP droplets were FRAP using confocal microscopy (NIKON A1RS) for a total of 5 min. Defined regions were photobleached and monitored at the 488 nm wavelength fluorescence intensities; in the NIH3T3 living cell, the TPPP-IDRs-FusionRed/Citrine-PixDE bundled MTs were FRAP and monitored at the 561 nm wavelength fluorescence intensities in every 5 s intervals for a total of 2 min. Recovery fluorescence intensities were recorded for the indicated time, then quantified, and normalized by using Fiji.

### Negative stain electron microscopy

The GFP-TPPP FL, GFP-TPPPCORE, and GFP-SynMAPs were mixed with unlabeled GMPCPP-stabilized MTs in the 1xBRB80 buffer for 5 min at room temperature. The mixed samples were diluted with 1xBRB80 to reduce unbundled MTs in the background, and a 3 μl mixture was spotted on glow-discharged grids (Electron Microscopy Sciences, CF400-Cu), then stained with 2% uranyl acetate. Images were collected at a magnification of 45,000 using 120 kV Talos L120C at UW-Madison Cryo-EM Research Center.

### Construction of cell lines using lentiviral transduction

Optogenetic Cry2WT expressing constructs and FRB-IDRs-PixDE/TPPP variants-FKBP constructs were produced using lentivirus. Pantropic VSV-G pseudotyped lentivirus was produced by transfecting 293T cells (ATCC CRL-3216) with a pLV-EF1a-IRES or pTwist-SFFV transgene expression vector and the viral packaging plasmids psPAX2 and pMD2.G using Fugene HD (Promega #E2312). Viral production was performed in 6-well tissue culture–treated plates (Corning 3335). Viral supernatants were collected 3 days after transfection and passed through a 0.45-mm filter to remove cell debris and then incubated with NIH 3T3 cells containing polybrene 8 μg/ml. Viral medium was replaced with normal growth medium 24 h after infection. Following transduction, unsorted populations of cells spanned a wide range of expression levels that were useful for exploring synMAP behavior across different component concentrations. To facilitate the comparison of expression-matched cells, after 3 days, these cells were sorted on an FACSAria cell sorter (BD Biosciences) based on fluorescent protein expression levels for further analysis.

### Live cell imaging

Cells were plated on 0.17 ± 5 μm 24 well glass bottom plate (Cellvis) in the FluoroBrite DMEM (Thermo Fisher Scientific A1896701) overnight. All live cell imaging was performed using 60X oil immersion objective (NA 1.4) on a Nikon Ti-Eclipse with a temperature stage at 37 °C and supplied with 5% CO2. NIH3T3 cells were imaged using four wavelengths (405 nm for tagBFP; 488 nm for Citrine; 561 nm for mCherry or FusionRed; 674 nm for SiR700-Tubulin Kit, #CY-SC014, Cytoskeleton Inc).

### OptoDroplet assay

The OptoTPPP was designed based on the strategy from the original OptoDroplets study (38). NIH3T3 cells were transfected with OptoFUS control constructs and OptoTPPP variants constructs to test. Cells were plated on 0.17 ± 5 μm 24 well glass bottom plate (Cellvis) in FluoroBrite DMEM (Thermo Fisher Scientific A1896701) for imaging. Cluster formation was induced using Nikon Ti-Eclipse with a Mightex Polygon digital micromirror device (DMD) at 475 nm wavelength light. The clusters and MTs were imaged using two wavelengths (561 nm for mCherry; 647 nm for SiR700-tubulin).

To quantify the cluster ratio in cells containing clusters, total cluster intensity (*I*_*C*_) was divided by the whole cell's total fluorescence intensity (*I*_*W*_) adapted from a previous study (68). The cluster disassembly rate was based on quantifying cluster number following stimulation over time normalized to the initial post-stimulation number. Curves were quantified by fitting them to a one-phase decay equation.

### Quantification of MT bundles in living cells

MT bundles were defined based on the fluorescence intensity of SiR-tubulin using a previously established method (65, 66). In each cell, we identified the region with the highest MT bundling (often near the cell center of cells) and measured the SiR-tubulin fluorescence intensity along straight-line segments of approximately 5 μm in length. This intensity was then normalized by dividing it by the fluorescence intensity of single MTs within the same cell, yielding the normalized MT bundling intensity (69, 70). All the quantification analyses of the fluorescence images were performed using Fiji.

### Nocodazole treatments

For MT depolymerization, nocodazole (30 μM) was added to the culture media for 6 h. Then, the NIH3T3 cells were washed with cold FluoroBrite medium directly at the microscope with temperature stage at 37 °C and supplied with 5% CO2. The heated stage slowly raised the temperature. The regrowth MT images and aster formation were captured using Nikon Ti-Eclipse at the wavelengths indicated in the figures.

### Statistical analysis

Statistical analyses were calculated using Prism (version 9.0c). Figure legends detail the n values and error bars for each experiment. Statistical differences for single-MT binding assay (Fig. S2, *C* and *E*) and cluster formation assay (Fig. 3*C*) were determined by Student’s two-tailed *t* test; ∗*p* < 0.05, ∗∗*p* < 0.01, ∗∗∗*p* < 0.001 and ∗∗∗∗*p* < 0.0001.

## Data availability

The authors declare that the data supporting the findings of this study are available within the manuscript and from the authors on request. Plasmids used in this study will be provided upon request.

## Supporting information

This article contains supporting information.

## Conflict of interest

The authors declare that they have no conflicts of interest with the contents of this article.
